# A Mini Review of Reused End-of-Life Reverse Osmosis (EoL RO) Membranes

**DOI:** 10.3390/membranes15070217

**Published:** 2025-07-21

**Authors:** Anissa Somrani, Kholoud Abohelal, Maxime Pontié

**Affiliations:** 1 Physics Department, College of Sciences, Jouf University, Sakaka P.O. Box 2014, Saudi Arabia; kkabohlal@ju.edu.sa; 2 Group of Analysis & Processes, Faculty of Sciences, University of Angers, CEDEX 01, 49045 Angers, France; maxime.pontie@univ-angers.fr

**Keywords:** EoL RO membranes, fouling, cleaning technologies, recycling applications, circular economy

## Abstract

As sensitive parts of the water treatment process, reverse osmosis (RO) membranes are the most important for desalination and wastewater treatment. But the performance of RO membranes deteriorates over time due to fouling, necessitating frequent replacements. One of the environmental challenges is the disposal of End-of-Life (EoL) RO membranes, which are made of non-biodegradable polymers. The reuse of EoL membranes as a sustainable approach for waste saving and resource efficiency has recently attracted considerable attention. The present work provides a comprehensive overview of the strategies for reusing EoL RO membranes as sustainable alternatives to conventional disposal methods. Furthermore, the fundamental principles of RO technology, the primary types and impacts of membrane fouling, and advanced cleaning and regeneration techniques are discussed. The conversion of EoL membranes into nanofiltration (NF), ultrafiltration (UF), and forward osmosis (FO) membranes is also covered in this review, as well as their uses in brackish water desalination, dye/salt separation, groundwater treatment, and household wastewater reuse. Environmental and economic benefits, as well as technical, social, and regulatory challenges, are also discussed. Finally, the review highlights innovative approaches and future directions for incorporating EoL membrane reuse into circular economy models, outlining its potential to improve sustainability and reduce operational costs in water treatment systems.

## 1. Introduction

The principal fouling types that reduce the efficiency of RO membranes are colloidal fouling, organic fouling, biofouling, and inorganic scaling. This contributes to a decrease in water flux, higher operating costs, and a shorter membrane lifespan [1,2,3,4]. Biofouling is especially problematic because dead microorganisms and their extracellular products can remain in the membrane channels after disinfection, causing persistent clogging that is very difficult to remove, even with intensive chemical cleaning [2,3,5]. Regardless of the state of the active layer, this clogging can make the membrane module unusable by limiting water flow. Additionally, the active polyamide layer of the membrane may be damaged by frequent or aggressive chemical cleaning, which is frequently used to avoid fouling and reduce the membrane’s selectivity and salt rejection [6,7]. The membrane may still work for less demanding procedures like nanofiltration (NF) or ultrafiltration (UF), where reduced selectivity is acceptable, even though such damage may render it unsuitable for high-purity RO applications [8,9]. As long as the channels are maintained sufficiently broad to allow water passage, it is viable to reuse damaged RO membranes under certain circumstances. Regardless of the state of the active layer, water cannot flow through the membrane effectively if the channels are severely blocked, particularly by biofouling or inorganic scaling [4,5]. In such cases, the membrane cannot be used again for any kind of filtration. Consequently, the degree of channel blockage as opposed to merely active layer degradation determines the possibility of repurposing used RO membranes.

By 2025, over 2 million end-of-life RO membrane modules are expected to be generated worldwide each year [10]. According to current estimates, over 1.5 million waste RO membrane modules are produced annually on a global scale [9]. Each year, thousands of tonnes of this waste are disposed of frequently in landfills [11].While landfill and incineration remain the most common disposal methods [12,13], chemical recycling and the repurposing of EoL RO membranes are becoming more popular as sustainable alternatives [14,15,16]. These methods reduce environmental impact, extend membrane life, and promote the circular economy principles in water treatment. Senán-Salinas et al. [17] examined the cost-effectiveness analysis at the pilot scale of end-of-life reverse osmosis recycling membranes. The study highlighted that approximately 14,000 tonnes of EoL RO membrane waste are landfilled annually, but it does not provide specific cost data for landfill or incineration. Recycling costs are detailed and appear EUR 25.9–73.75 per module, but direct cost comparisons to landfill or incineration are not available in this study [17]. Exploring the reuse of end-of-life (EoL) reverse osmosis (RO) membranes facilitates advancements towards sustainability goals in wastewater treatment. Reusable EoL membranes are generally viewed as spent due to fouling and performance decline; however, they possess remarkable capabilities that can alleviate both ecological and economic concerns pertaining to membrane disposal. Bioorganic materials, as well as inorganic compounds such as silica, lead to chemically irreversible fouling engulfing membranes and forcing replacement to restore function. The replacement of membranes has been identified as one of the most costly factors pertaining to RO systems [18,19,20]. Regardless of challenges, new methods have been created that overcome these hurdles towards successful membrane recycling.

Recycling EoL RO membranes entails converting them into alternative filtration systems, like ultrafiltration (UF) or nanofiltration (NF) membranes, which have a variety of uses, such as dye/salt separation and the treatment of domestic wastewater [15,21,22]. These recycled membranes are appropriate for decentralized water treatment systems, especially in isolated regions and developing countries, due to their enhanced permeability and fouling resistance [21]. Furthermore, it has been demonstrated that chemical treatments like chlorination increase the water permeability and preserve the selectivity of RO membranes, extending their lifespan and lowering the frequency of replacements [23].

By decreasing waste, the use of recycled EoL RO membranes in wastewater treatment not only promotes a circular economy but also provides an affordable water purification option. These membranes can be successfully incorporated into current water treatment infrastructures by addressing the fouling mechanisms and streamlining the recycling procedures, offering a sustainable substitute for conventional disposal techniques [5,12]. Overall, reusing EoL RO membranes has a lot of potential to improve wastewater treatment technologies and encourage environmental sustainability.

## 2. Basic Principles of Reverse Osmosis (RO) Membrane

Driven by the transmembrane hydraulic pressure difference, reverse osmosis (RO) is a popular membrane technology for water treatment and desalination. Its effectiveness in removing dissolved salts and other contaminants from water is well-known. Basic principles of the RO membrane are discussed individually below.

### 2.1. Membrane Structure and Materials

Polyamide thin-film composites (PA-TFC), which have high water permeability and salt rejection, are commonly used to make RO membranes. These membranes, which offer superior separation performance and mechanical strength, are made up of a thin layer of polyamide, a porous substrate, and a nonwoven fabric support [24,25,26].

### 2.2. Transport Mechanisms

The transport of salt and water in RO membranes is frequently described by the solution-diffusion (SD) model. Diffusion through the membrane matrix occurs after solutes and water are partitioned into the membrane in this model. But by taking into account the interactions between water, salt ions, and the membrane, as well as the friction between these elements, the solution-friction (SF) model provides a more thorough understanding [27,28].

### 2.3. Pressure-Driven Membrane Process

In order to force water through the membrane while keeping solutes in place, RO applies pressure to overcome the natural osmotic pressure. The hydraulic pressure gradient across the membrane is crucial to the process [28,29].

### 2.4. Challenges and Improvements

In RO systems, membrane fouling and chlorine degradation are major problems. To improve membrane performance and resistance to fouling and chlorine, surface modifications and sophisticated fabrication techniques, like co-solvent interfacial polymerization, have been developed [6,24,25].

A key technology for desalinating water, reverse osmosis relies on pressure-driven membrane processes and has effective separation capabilities. Its performance is still being enhanced by developments in membrane materials and transport models, which tackle issues like fouling and chlorine resistance. Gaining an understanding of these fundamental ideas is crucial to improving RO systems and increasing their use.

## 3. End-of-Life (EoL) Reverse Osmosis (RO) Membranes

The disposal of end-of-life (EoL) membranes, especially those utilized in reverse osmosis (RO) and other filtration processes, poses serious environmental and financial problems. To solve these problems, recent studies have concentrated on recycling and sustainable management techniques.

### 3.1. Recycling and Transformation Strategies

Blockage of channels by sediments such as organic matter, biofilms, and inorganic particles is the main problem with RO membrane systems. Long-term use makes it difficult to remove them, reducing performance. For less demanding applications, membranes at the end of their life can be recycled; however, thorough cleaning is necessary. Reversible fouling is effectively removed by physical cleaning techniques such as air sparging and osmotic backwashing. For example, Motsa et al. [30] used a cellulose triacetate(CTA) forward osmosis membrane to evaluate how well an online osmotic backwash (OsBW) restored membrane flux following fouling by seawater-like feed. Although some fouling residues remained in the support layer, the results demonstrated that increased permeation rates and cross-flow velocities enhanced cleaning efficiency, recovering over 90% of flux [30]. Similarly, Saleh et al. [31] investigated using air sparging to improve reverse osmosis (RO) performance, with an emphasis on increasing permeate flux and salt rejection under various operating conditions [31]. Ecological substitutes are provided by new methods such as vibrational cleaning and micro/nanobubbles [32].

To remove microbial, inorganic, and organic soils, chemical cleaning is essential. A wide range of fouling agents, such as proteins, polysaccharides, and metals, can be removed and up to 45–70% of membrane flux can be restored through optimized procedures combining alkaline agents (NaOH), acids (HCl, citric acid), surfactants (Sodium Dodecyl Sulfate (SDS)), and chelating agents (EDTA) [33,34,35]. Compared to traditional agents, urea and free ammonia demonstrated superior removal of biofilm and organic matter; urea is also environmentally friendly and delays the appearance of new clogging [36,37].

By combining chemical agents with physical agitation (such as ultrasound), hybrid techniques, such as ultrasound-assisted cleaning, improve soil detachment and removal, especially for complex soil layers and stubborn biofilms. Hybrid methods can further improve membrane efficiency and recovery [32]. Additionally, hybrid approaches and optimized protocols that optimize both chemical and physical parameters (sequence, concentration, and temperature) achieve the highest cleaning efficiencies and longest membrane life [38].

There are several ways to convert EoL RO membranes into NF membranes. One method produces membranes with enhanced fouling resistance and high dye/salt separation efficiency by oxidatively treating and co-depositing polydopamine and polyethylene glycol [22]. Another technique achieves competitive performance with commercial NF membranes by using polyelectrolyte layer-by-layer deposition [39].

When metal ions are present, oxidants such as NaOCl can accelerate the breakdown of the polyamide layer in EoL membranes, transforming them into membranes that resemble ultrafiltration. This process allows for control over the membrane’s molecular weight cut-off, enhancing their reuse potential [40].

Downcycling is a recent membrane recycling technique that involves converting end-of-life (EOL) high-pressure membranes, such as RO, into lower-pressure NF or UF membranes through regulated chemical treatments, such as NaOCl oxidation, frequently combined with an alkaline pre-treatment to control fouling [12,41,42]. Upcycling is the process of converting low-pressure or fouled membranes into new, high-performance membranes. For example, interfacial polymerization [12,41,43] can be used to create thin-film composite polyamide membranes from biopolymer-fouled microfiltration substrates. These closed-loop methods have substantial economic advantages and can lessen environmental effects by up to 22–27%. Both approaches contribute to the closure of the membrane technology loop and increase the sustainable use of EOL membranes [12,41,42,43].

### 3.2. Balancing Ecology and Economy

By eliminating the need to produce new membranes, a closed-loop recycling approach for EoL membranes can offer financial benefits in addition to a significant reduction in environmental effects and CO_2_ emissions [41]. Life cycle assessments that emphasize recycling smaller environmental impact than conventional disposal techniques lend support to this strategy [44].

EoL membrane conversion and recycling not only cut waste but also open up business opportunities. For example, it is both economically and environmentally advantageous to convert EoL RO membranes to ultrafiltration membranes, which could lead to the creation of jobs and other social benefits [45].

### 3.3. Regeneration Techniques

EoL membrane regeneration can improve antifouling qualities and restore permeability through solvent treatments and chemical cleaning. Membrane service life in wastewater treatment applications can be increased by employing methods like triethyl phosphate or green solvents, which have been demonstrated to efficiently remove irrecoverable foulants [45,46,47].

### 3.4. Challenges and Future Directions

Even though existing techniques are promising, more optimization is required to improve recycling processes’ scalability and efficiency. This entails tackling the environmental effects of long-distance transportation and refining the characterization methods for recycled membranes [44].

For membrane technology to be sustainable, EoL membrane recycling must be incorporated into a circular economy framework. To improve these procedures and guarantee their financial sustainability and environmental sustainability, more research and development is required [12,48].

There are major financial and environmental advantages to managing EoL membranes sustainably through recycling and regeneration. These strategies not only lessen the adverse effects of disposing of membranes, but they also help membrane technology advance circular economy principles.

## 4. Fouling of RO Membranes

Fouling severely reduces the effectiveness of reverse osmosis (RO) membranes, which are essential for wastewater treatment and water desalination. Fouling is a significant obstacle to the sustainable use of RO technology since it results in decreased permeate flux, decreased salt rejection, higher energy requirements, and a shorter membrane lifespan [4,49,50].

### 4.1. Types of Fouling and the Effective Removal Methods

There are different forms of fouling, depending on the foulant. These consist of colloidal fouling, organic fouling, inorganic fouling, and bio-fouling. The buildup of microorganisms on the membrane surface results in biofilms that impede water flow, which is the cause of biofouling [3,49]. Proteins and polysaccharides, which are common in wastewater and can drastically impair membrane performance, can deposit and cause organic fouling [33,51]. Hard scales may form on the membrane surface as a result of the precipitation of inorganic salts such as magnesium and calcium carbonate, a process known as inorganic fouling (scaling) [5,52]. The buildup of colloidal particles causes colloidal fouling, which can obstruct membrane pores and decrease permeability [3,49]. Effective removal of these contaminants is crucial for safe handling, recycling, or disposal of spent membranes. In Table 1, typical contaminants contained in used (RO) membranes and their effective removal methods are summarized.

### 4.2. Mechanisms and Implications

Complex interactions between membrane materials and water pollutants cause foulants to build up on the membrane’s surface or inside its pores. This buildup eventually lowers the quality and amount of desalinated water by decreasing permeate flux, raising operating pressure, and requiring more frequent chemical cleaning [33,50].

### 4.3. Mitigation Strategies

Several methods have been put forth to lessen membrane fouling. The characteristics of the membrane and the feed solution determine how applicable these techniques are. Among these methods are the following:

Pre-treatment: Before feed water reaches the RO membranes, it must undergo effective pre-treatment procedures like coagulation and ultrafiltration to lower its fouling potential [4,52].

Chemical cleaning: By removing both organic and inorganic foulants, improved acid-base solutions and other optimized chemical cleaning techniques can greatly improve membrane performance [33].

Surface modification: By reducing foulant adhesion, improving the hydrophilicity and smoothness of membrane surfaces can minimize fouling [3,57].

Novel membrane materials: The goal of research into novel materials and synthesis techniques is to create RO membranes with enhanced antifouling capabilities and extended lifespans [57,58].

### 4.4. Limitations and Future Perspectives

There are still difficulties in completely comprehending and managing fouling processes, even with improvements in fouling mitigation. In order to improve the antifouling properties of RO membranes, future research should concentrate on creating more precise prediction and diagnostic methods, as well as investigating novel materials and technologies [50,57]. Furthermore, encouraging a circular economy in RO desalination requires addressing the end-of-life management of fouled membranes [58].

## 5. Challenges in Reusing EoL RO Membranes

Reusing end-of-life (EoL) reverse osmosis (RO) membranes poses a number of difficulties, chiefly because of technical, financial, and environmental considerations.

### 5.1. Environmental and Economic Challenges

Disposal practices: Existing disposal techniques, like incineration and landfilling, are unsustainable and raise environmental issues [12,17].

Cost-effectiveness: Recycling procedures may be expensive. For example, both the active system (AS) and the passive system (PS) have substantial costs, although the PS is less expensive [17].

### 5.2. Technical Challenges

Membrane degradation: EoL RO membranes frequently experience fouling and degradation, making it more difficult to reuse them or convert them into other membrane types, such as ultrafiltration (UF) or nanofiltration (NF) [42,59].

Foulant removal: Regenerating membranes depends on the efficient removal of irrecoverable foulants. Although methods like green solvent cleaning and alkaline pre-treatment have been investigated, they need to be handled and optimized carefully [42,46].

Performance limitations: Some conversions may not be technically competitive due to decreased permeability and performance when EoL RO membranes are converted into other membrane types [17,60].

### 5.3. Social and Regulatory Challenges

Environmental impact, cost, technical constraints, and social concerns are some of the major obstacles to reusing EoL RO membranes. To overcome these obstacles and increase the viability of reusing these membranes, creative recycling technologies, economical procedures, and environmentally friendly practices are needed.

Recycling and conversion have the potential to generate employment, but the social advantages must be weighed against environmental sustainability and economic feasibility [61].

## 6. Technologies and Methods for Reusing EoL RO Membranes

Because of their buildup in landfills and incineration, end-of-life (EoL) reverse osmosis (RO) membrane disposal presents serious environmental issues. In order to reduce environmental effects and advance sustainability, recent research has concentrated on recycling and reusing these membranes.

### 6.1. Conversion to Nanofiltration (NF) and Ultrafiltration (UF) Membranes

Polyelectrolyte layer-by-layer deposition: By depositing polyelectrolyte multilayers, EoL RO membranes can be transformed into nanofiltration (NF) membranes. This method improves the salt rejection capabilities of the membranes, achieving up to 98% rejection of MgSO_4_, making them competitive with commercial NF membranes [39].

Treatment with alkali and NaClO: EoL RO membranes can be downcycled into NF-like or UF-like membranes by an alkaline pre-treatment and exposure to sodium hypochlorite (NaOCl). Membranes with adequate filtration and antifouling performance are produced by this process, which also efficiently eliminates fouling layers and breaks down the polyamide layer [42].

Chemical oxidation for UF Conversion: Using NaOCl, EoL RO membranes can be chemically oxidized to create ultrafiltration (UF) membranes. By increasing permeability and decreasing salinity rejection, this process qualifies the membranes for use in point-of-use water treatment applications [21].

### 6.2. Direct Reuse and Regeneration

Direct reuse for brackish water desalination: EoL seawater RO membranes have NaCl rejection rates of 84–92%, which are on par with commercial membranes, and can be used directly for brackish water desalination. This strategy presents a viable way to lessen the negative effects on the environment while assisting the desalination sector [60].

New Uses and Regeneration: EoL RO membranes can be used for new uses at reduced treatment rates or regenerated through cleaning treatments. Conversion into NF, UF, or microfiltration (MF) membranes is one way to increase their usefulness and cut down on waste [62].

### 6.3. Novel Transformation Techniques

Dye/Salt separation: This new technique uses oxidative treatment and polydopamine and polyethylene glycol co-deposition to convert EoL RO membranes into loose NF membranes. This method provides a sustainable way to treat wastewater contaminated with dyes by improving permeability and the efficiency of dye/salt separation [22].

Forward osmosis membranes: Recycling EoL RO membranes into forward osmosis (FO) membranes is a creative approach that helps the membrane technology industry move toward a circular economy by valuing a sizable amount of the membrane area and plastic components [63].

### 6.4. Environmental and Socioeconomic Considerations

Evaluations of the socioeconomic and environmental effects of recycling EoL RO membranes have shown the advantages of conversion to UF membranes and direct reuse. These alternatives offer financial benefits and the possibility of job creation, and they are more environmentally friendly than conventional disposal techniques [61].

EoL RO membrane recycling and reuse offer workable answers to the environmental problems posed by their disposal. Numerous approaches, such as direct reuse, conversion to NF and UF membranes, and innovative transformation techniques, present encouraging paths toward sustainable membrane management. These strategies improve the water treatment processes’ environmental and financial sustainability in addition to reducing waste.

## 7. Applications of Reused EoL RO Membranes

One promising strategy to improve sustainability and lessen environmental impact is the reuse of end-of-life (EoL) reverse osmosis (RO) membranes in wastewater treatment and brackish water desalination. There are several uses for these membranes, which have advantages for the environment and the economy.

### 7.1. Groundwater and Domestic Wastewater Treatment

In comparison to conventional RO systems, EoL RO membranes can be recycled into ultrafiltration (UF) membranes for point-of-use applications, which effectively treat groundwater with less energy [13]. They can also be used to treat domestic wastewater, meeting local drinking water and irrigation standards and achieving high rejection rates for contaminants [15].

### 7.2. Dye/Salt Separation

Transformed EoL RO membranes, which have high permeability and superior fouling resistance, can be utilized for effective dye/salt separation in wastewater. To improve their hydrophilicity and separation capabilities, this transformation involves coating and oxidative treatment procedures [22].

### 7.3. Forward Osmosis (FO) Technology

EoL RO membranes offer competitive wastewater treatment performance when modified for forward osmosis applications. Cleaning and interfacial polymerization techniques are used in this adaptation to improve the characteristics of the membrane [64].

### 7.4. Brackish Water Desalination

**Figure 1:**
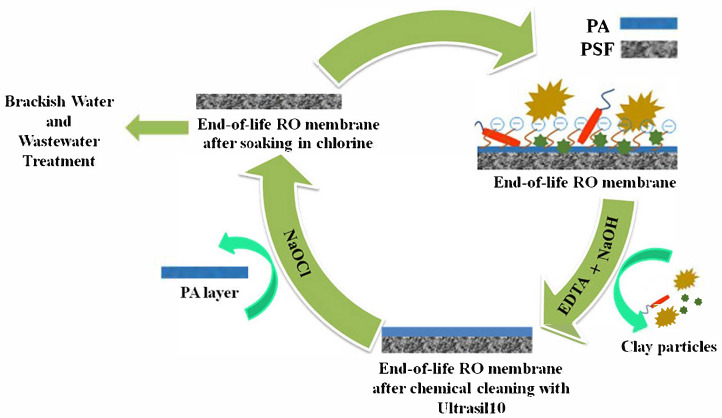
Schematic diagram of the potential chemistry between Ultrasil10 and chlorine with the polyamide layer of the EoL RO membrane [65].

EoL seawater RO membranes have salt rejection rates that are on par with those of commercial nanofiltration membranes, making them suitable for direct reuse in brackish water desalination. The environmental impact of disposing of membranes is lessened by this reuse [61]. Somrani et al. [65] explored the use of end-of-life seawater reverse osmosis (RO) membranes for treating brackish water and industrial effluent. To treat the membrane for reuse, the aged membrane was first treated with Ultrasil10 and then cleaned with a Cl_2_ solution. Figure 1 shows the potential chemical interactions between Ultrasil10 and chlorine with the polyamide layer of the EoL RO membrane. The elimination of fouling during the chemical cleaning procedure and the mechanical or chemical deterioration of the polyamide layer brought on by oxidative exposure are two important mechanisms highlighted in the schematic [65].

End-of-life RO membranes that have been cleaned show improved hydraulic permeability (1.97 L·h^−1^·m^−2^·bar^−1^) and an 85% salt rejection for brackish water (6 g/L NaCl). These membranes successfully decreased brackish water conductivity to below 1000 µS/cm at 10 bars, meeting Tunisian drinking water requirements (300–2500 µS/cm) with a molecular weight limit of 86 Da. They also showed excellent efficacy in treating industrial effluents, attaining a conductivity of 180 µS/cm and turbidity levels below 2 NTU. Lower operating pressures allowed these membranes to function similarly to new NF membranes sold commercially and offer affordable, environmentally friendly alternatives, confirming their promise for repurposing in brackish water and wastewater treatment applications [65].

### 7.5. Regeneration and Upcycling

The permeability and antifouling properties of EoL membranes can be restored by hydrophilic modification and chemical cleaning. This strategy lowers expenses and carbon emissions while promoting sustainable wastewater treatment [45,66].

One sustainable way to address membrane disposal issues is to reuse EoL RO membranes in wastewater treatment. These membranes can be efficiently modified for a number of uses, such as forward osmosis, dye/salt separation, and groundwater treatment, all of which support resource efficiency and environmental sustainability.

### 7.6. Advantages and Disadvantages of Different Approaches of EoL RO Membranes Application

There are major financial and environmental advantages to recycling and reusing EoL RO membranes, which are being actively developed in the water, industrial, and construction sectors. Performance, lifespan, and broader adoption optimization still present challenges, but new applications and continuous research are expanding the range of sustainable solutions. The advantages and disadvantages of different approaches to EoL RO membranes applications are reported in Table 2.

## 8. Future Prospects and Innovations of EoL RO Membrane Reuse

End-of-life (EoL) reverse osmosis (RO) membrane recycling and reuse offer substantial prospects for both economic viability and environmental sustainability. Since disposing of these membranes in landfills or burning them presents environmental problems, creative ways to reuse them are being investigated.

### 8.1. Recycling and Conversion Strategies

Direct reuse and conversion: EoL RO membranes can be transformed into other membrane types, like ultrafiltration (UF) and nanofiltration (NF), or they can be used directly again. Numerous techniques, such as chemical treatments and layer-by-layer deposition of polyelectrolytes, can accomplish this conversion, improving the permeability and rejection capabilities of the membranes [39,60,62].

Chemical treatments: One popular technique for altering EoL RO membranes is the chemical oxidation of sodium hypochlorite (NaOCl). By improving permeability and changing rejection characteristics, this treatment can successfully transform RO membranes into UF membranes, which makes them appropriate for point-of-use water treatment applications [21,42].

### 8.2. Environmental and Economic Benefits

Sustainability and circular economy: Recycling EoL RO membranes promotes material reuse and waste reduction, which is consistent with the principles of the circular economy. By lowering the need for new membrane production, this strategy not only reduces the negative effects on the environment but also has financial advantages [22,44,61].

Life cycle assessment: Research has demonstrated that recycling EoL RO membranes can have a major positive impact on the environment, especially in areas with large desalination capacity. The potential for lower greenhouse gas emissions and resource conservation is highlighted by the life cycle assessment of these processes [44,61].

### 8.3. Current Challenges and Outlook

Fouling and material degradation: Eliminating fouling and the polyamide layer’s deterioration are two major obstacles in the recycling of EoL RO membranes. For the membranes to function well and last in new applications, efficient cleaning and treatment techniques are necessary [42,59].

Technological developments: To maximize recycling procedures and enhance the functionality of converted membranes, more research and development is required. The membrane qualities can be improved by advancements in coating and chemical treatment technologies, increasing their competitiveness with commercial goods [22,59].

EoL RO membrane recycling and reuse present encouraging opportunities for environmentally friendly water treatment methods. These strategies can greatly improve the water treatment industry’s economic efficiency and environmental sustainability by tackling the problems of fouling and material degradation and utilizing technology breakthroughs.

## 9. Conclusions

Reusing End-of-Life (EoL) RO membranes offers a chance to cut waste and enhance the sustainability of membrane-based filtration systems for wastewater treatment. Recent developments in cleaning technologies, surface modification, and membrane materials hold promise for enhancing the performance of repurposed membranes, even though fouling and cleaning issues still exist. Realizing the full potential of recycled EoL RO membranes in wastewater treatment will require ongoing research into sustainable materials and circular economy models.

## Figures and Tables

**Table 1:** Typical contaminants contained in used (RO) membranes, and the effective removal methods.

Contaminant Type	Effective Removal Methods	References
Organic Contaminants (proteins, polysaccharides, and microbial metabolites)	Optimized chemical cleaning, Membrane Bioreactor (MBR) + RO Treatment	[33,53,54]
Inorganic Contaminants (scaling agents: calcium, magnesium, iron, and other mineral deposits)	Acid–base cleaning (with citric acid, NaCl)	[33,55]
Heavy Metals (arsenic and other toxic metals)	RO/NF filtration, pre-treatment	[55,56]
Pharmaceuticals	Membrane Bioreactor (MBR) + RO, high-flux RO/NF	[53,54,56]

**Table 2:** Advantages and disadvantages of different approaches to EoL RO membranes applications.

Approach/Reuse Application	Sector/Use Case	Advantages	Disadvantages/Challenges	References
Chemical conversion to UF/NF	Water treatment	High cost savings, environmental benefits	Shorter lifespan than new membranes	[39,67,68]
Oleochemical wastewater recycling	Industrial water reuse	Effective COD/TDS * reduction	Biofouling, requires frequent cleaning	[69]
Gray water reclamation	Domestic reuse	Low turbidity, cost-effective, landfill reduction	Limited pilot data, fouling potential	[70]
Membrane distillation (MD) support	Desalination	High salt rejection, extends module life	Support structure limitations	[71]
Forward osmosis (FO)	Wastewater treatment	Comparable to commercial FO membranes	Requires adequate cleaning/modification	[64]
Concrete additive	Construction	Improved strength, sustainability	Increased porosity at high loadings	[72]
Creative non-filtration uses	Various	Reduces landfill, versatile	Limited technical validation	[73]

* COD/TDS: Chemical Oxygen Demand and Total Dissolved Solids.

## Data Availability

The original contributions presented in this study are included in the article. Further inquiries can be directed to the corresponding author.
